# Octogenarians with chronic kidney disease in the nephrology clinic: Progressors vs. non-progressors

**DOI:** 10.3389/fneph.2023.1114486

**Published:** 2023-02-01

**Authors:** Aida Frías, Francisco Vargas, Justo Sandino, Raquel Berzal, Marta Rivero, Lucía Cordero, Teresa Cavero, Julián Segura, Florencio García, Eduardo Hernández, Eduardo Gutiérrez, Pilar Auñón, Irene Zamanillo, Julio Pascual, Enrique Morales

**Affiliations:** Department of Nephrology, Hospital 12 de Octubre, Madrid, Spain

**Keywords:** elderly, chronic kidney disease, albuminuria, estimated glomerular filtration rate, CKD-EPI

## Abstract

**Background:**

The current definition of chronic kidney disease applied to patients over the age of 80 has increased the number of referrals to Nephrology. However not all of these patients may benefit from its assessment. This study aims to analyze the evolution of ≥80 years old patients referred to Nephrology.

**Methods:**

Single-center study including patients ≥80 years old with eGFR <60 mL/min/1,73m2 who were referred to Nephrology consultation for the first time. Clinical and analytical parameters were collected retrospectively 12 months before the visit, and prospectively at baseline, and 12 and 24 months after the initial visit. We divided patients into two groups based on annual eGFR loss: progressors (>5 mL/min/1.73m2) and non-progressors (≤5 mL/min/1,73m2).

**Results:**

A total of 318 patients were included, mean age was 84,9 ± 4 (80-97) years. Baseline serum creatinine was 1,65 ± 0,62 mg/dL, eGRF 35 (28-42) mL/min/1,73, and albumin/creatinine ratio 36 (7-229) mg/g. 55,7% of the patients met the definition of progressor at baseline (initial-progressors), 26,3% were progressors after a 12-month follow-up and 13,4% after 24 months. 21,2% and 11,4% of initial-progressors met this definition at 12 and 24 month follow up. The main risk factor for progression was albuminuria. No relationship was found between the nephrologist intervention and the evolution of renal function among initial non-progressors.

**Conclusion:**

Elderly patients who have stable renal function at the time of referral will continue to have stable renal function over the subsequent 24 months and thus may not need to be referred to a nephrologist.

## 1 Introduction

Chronic kidney disease (CKD) has increased its prevalence among patients over 65 years old (1). In Spain, there are 4 million people affected, and it is estimated that 22% of people over 64 and 40% of those over 80 years old meet the criteria for diagnosis (2). Hypertension and diabetes mellitus (DM), common comorbidities in the general population, are the leading causes of CKD.

Several epidemiological studies of Nephrology societies have shown that known CKD is the tip of the iceberg of what is considered hidden kidney disease, which could lead to a worldwide epidemic (3, 4). This pathology represents a significant public health issue with tremendous socioeconomic impact. All this has led to increasing efforts for early CKD detection, and determination of estimated glomerular filtration rate (eGFR) in the routine analysis of General Practitioners (GP) consultations has been promoted.

Nevertheless, systematic determination of eGFR can lead to excessive referrals to the Nephrology department in elderly patients, and some points must be considered. For instance, GFR declines physiologically with age at an average rate of 0.8 to 1 mL/min/year, and most of the patients die due to cardiovascular diseases before CKD progresses (1, 5, 6).

This raises a debate about whether the reduction of GFR that occurs with age should be considered as “disease” or as “age-related decline” (1, 5, 7–10); and if a Nephrology specialist’s follow-up of the geriatric population represents an advantage for its evolution and prognosis compared to GP’s management alone (1, 5, 7, 11–13).

Therefore, it is essential to design screening strategies to select patients who may benefit from closer monitoring in order to avoid a healthcare system overload (5, 6, 9, 12, 13). According to the Spanish consensus document for the detection and management of CKD (14), patients >80 years with no evidence of progression and no significant albuminuria could remain under GP follow-up even with an eGFR <30 mL/min/1.73m2.

The aim of this study was to analyze the evolution of renal function and the risk factors for CKD progression on octogenarian patients with an eGFR <60 mL/min/1.73m2 referred to Nephrology consultations.

## 2 Methods

This is an observational study including patients ≥80 years old with an eGFR <60 mL/min/1.73m2 who were referred for the first time to the Nephrology department from GP or from other specialties between March 2017 and March 2019. The study was conducted at the Nephrology department of the University Hospital 12 de Octubre of Spain. The consulting nephrologist performs a comprehensive assessment of the patient.

Clinical and analytical parameters were collected retrospectively 12 months before the initial visit, and prospectively from baseline to 24 months after. Epidemiological data collected included age, sex, the reason for consultation, and several comorbidities such as DM, hypertension, dyslipidemia (DL), obesity, cardiovascular disease (including heart failure [HF], coronary heart disease, valvular heart disease, and arrhythmias such as atrial fibrillation, flutter, and atrioventricular block), peripheral vascular disease, lung disease, tumor disease or presence of a solitary kidney. The Charlson comorbidity index was also applied, which assigns a score according to the number of associated pathologies and relates it to the patient’s mortality risk.

Laboratory data included sCr, eGFR according to CKD-EPI formula (chosen because its easy applicability and widespread use) expressed in mL/min/1.73m2, albumin/creatinine ratio (ACR), 24-hour proteinuria, and sodium, potassium, calcium, phosphorous, and uric acid levels. All measurements were performed at stable condition as part of routine check-ups (not during admissions or intercurrent processes).

According to the definition of the Spanish consensus document for the detection and management of CKD (14), two study groups were established based on eGFR decrease 12 months before consultation and at the time of the first Nephrology evaluation. We considered as “initial progressors” those patients with an eGFR decline >5 mL/min/1.73 m2, and “initial non-progressors” those with an eGFR decline ≤5 mL/min/1.73 m2.

Evolution after 12 and 24 months from the time of the first consultation was analyzed globally and within the subgroups of initial progressors and initial non-progressors. Those with an eGFR decline >5 mL/min/1.73 m2 between the time of consultation and the subsequent 12 months were considered “progressors at 12 months”, and those with an eGFR drop ≤5 mL/min/1.73 m2, “non-progressors at 12 months”. We considered “progressors at 24 months” those with an eGFR decline >10 mL/min/1.73 m2 between the time of consultation and the subsequent 24 months (that is, those with a mean annual eGFR drop > 5 mL/min/1.73 m2), and as “non-progressors at 24 months” those with an eGRF decline ≤10 mL/min/1.73 m2.

A subanalysis of patients with very low eGFR (<30 mL/min/1.73m2) was also performed, which according to the aforementioned consensus could be non-referred under certain conditions.

Statistical analysis was performed with IBM SPS Statistics^®^ v25.0 program. Mean and standard deviation (SD) were used for homogeneous distribution measures and median and interquartile range (IQR) for those of asymmetric distribution. The Chi-square test was used for comparison of categorical variables, Student’s t test for comparison of categorical with quantitative of normal distribution variables and Mann-Whitney U test for comparison of categorical with quantitative of non-normal distribution variables. Logistic regression was used for multivariate (MV) analysis. Bilateral p <0.05 values were considered statistical significant. 

## 3 Results

### 3.1 Baseline characteristics

A total of 318 patients aged ≥80 years who attended the Nephrology department for the first time between March 2017 and March 2019 were analyzed. Baseline characteristics are described in **Table 1**. The majority (n=175, 55%) were women, and the mean age was 84.9 ± 4 (80–97) years. Mean sCr was 1.65 ± 0.62 mg/dL and median eGFR was 35 (28-42) mL/min/1.73. Median ACR was 36 (7-229) mg/g; in 13 patients (9.1%) albuminuria was >1000 mg/g. Significantly higher median baseline albuminuria was found in males (males 89 [19-478] mg/g, females 16 [4-105] mg/g, p<0.001).

**Table 1:** Baseline characteristics at the time of first Nephrology consultation (n=318).

Age (years), mean ± SD (range)		84.9 ± 4 (80-97)
Female, n (%)		175 (55)
Referral reason		
*Impaired renal function* n (%)		291 (91.5)
*Proteinuria* n (%)		8 (2.5)
*Hypertension* n (%)		6 (1.9)
*Anemia* n (%)		3 (0.9)
*Ionic alterations* n (%)		3 (0.9)
*Edema* n (%)		2 (0.6)
*Other*		5 (1.6)
Comorbidities		
*Hypertension* n (%)		285 (89.6)
*Dyslipidemia* n (%)		134 (42.1)
*Diabetes mellitus* n (%)		131 (41.2)
*Arrhythmia* n (%)		62 (19.5)
*Tumor disease* n (%)		62 (19.5)
*Ischemic heart disease* n (%)		60 (18.9)
*Heart failure* n (%)		30 (9.4)
*Lung disease* n (%)		27 (8.5)
*Valve disease* n (%)		24 (7.5)
*Peripheral vascular disease* n (%)		19 (6)
*Obesity*		15 (4.7)
*Solitary kidney* n (%)		11 (3.5)
Charlson Comorbidity Index		
*Mean ± SD*		6.99 ± 1.7
*0-4 points.* n (%)		30 (9.4)
*5-8 points*, n (%)		228 (71.7)
*9-12 points*, n (%)		60 (18.9)
Most common drugs		
*ACEI/ARB* n (%)		246 (77.4)
*Statins* n (%)		184 (57.9)
Diabetic patients (n=131)	*Oral antidiabetic agents* n (%)	82 (62.6)
	*Insulin* n (%)	12 (9.2)
	*Insulin + Oral antidiabetic agents* n (%)	37 (28.2)
Laboratory parameters		
*Serum creatinine* (mg/dL), mean ± SD		1.65 ± 0.62
*Estimated Glomerular Filtration Rate* (mL/min/1.73 m^2^), median (RIQ)		35 (28-42)
*Albumin-creatinine ratio* (mg/g), median (IQR)		36 (7-229)
*24h proteinuria* (g/24h), median (IQR)		0.26 (0.13-0.69)
*Hemoglobin* (g/dL), mean ± SD		12.4 ± 1.7
*Sodium* (mEq/L), mean ± SD		142 ± 3.2
*Potassium* (mEq/L), mean ± SD		4.9 ± 0.6
*Calcium* (mg/dL), mean ± SD		9.6 ± 0.6
*Phosphorus* (mg/dL), mean ± SD		3.4 ± 0.6
*Uric acid* (mg/dL), mean ± SD		7.1 ± 1.8

ACEI, Angiotensin converting enzyme inhibitors; ARB, Angiotensin receptor blockers; SD, Standard deviation; IQR, interquartile range.

The main referral reason to the nephrologist was impaired renal function (n=291, 91.5%), with no significant differences between initial-progressors (n=161, 91%), and initial non-progressors (n=130, 92.2%), p=0.694. The second most frequent reason was proteinuria (n=8, 2.5%) (**Table 1**), and no differences between theses groups were found: initial-progressors n=4 (2.3%) vs initial non-progressors n=4 (2.8%), p=0.744). The most frequent comorbidity was hypertension (n=285, 89.6%).

The most common suspected diagnosis was isolated nephroangiosclerosis (NAE) (46.2%), followed by a combination of NAE with diabetic kidney disease (DKD) or diabetic nephropathy (DN) (18.6%). The etiological diagnosis of nephropathy was based on the exclusion of other pathologies and was justified according to the clinical criteria of the nephrologist of the department. The renal biopsy was not performed in the vast majority of patients due to age and fragility.

Regarding the therapeutic/diagnostic nephrologist decision, 39.6% continued with the same treatment. Diuretics and/or other antihypertensive drugs were reduced or discontinued in 25.5%, while they were increased in 8.5%. In 26.4%, other actions were carried out, such as adjusting anemia treatment or requesting imaging tests (**Table 2**).

**Table 2:** Suspected diagnosis and interventions after first Nephrology consultation.

Diagnosis	
*NAE*, n (%)	147 (46.2%)
*DKD/DN and NAE*, n (%)	59 (18.6)
*DKD/DN*, n (%)	15 (4.7%)
*Chronic interstitial nephritis*, n (%)	11 (3.5%)
*Glomerular disease*, n (%)	3 (0.9)
*Other*, n (%)	18 (5.7)
*Not available*, n (%)	65 (20.4)
Intervention	
*None*, n (%)	126 (39.6)
*Reduce diuretics and/or other antihypertensive drugs*, n (%)	81 (25.5)
*Increase diuretics and/or other antihypertensive drugs*, n (%)	27 (8.5)
*Other*, n (%)	84 (26.4)

DN, diabetic nephropathy; DKD, diabetic kidney disease; NAE, nephroangiosclerosis.

### 3.2 Evolution of renal function

eGFR decreased by a median of 6 mL/min/1.73m2 before Nephrology assessment (eGFR 35 [28-42] mL/min/1.73m2). However, it remained in similar values throughout the rest of the follow-up (37 [28-46] and 35 [26-44] mL/min/1.73m2 at 12 and 24 months, respectively) (**Table 3**).

**Table 3:** Evolution of renal function in all patients.

		Month -12	Baseline (first Nephrology consultation)	Month +12	Month +24
sCr (mg/dL), mean ± SD	Global	1.4 ± 0.43	1.65 ± 0.62	1.66 ± 0.7	1.74 ± 0.92
	IP	1.27 ± 0.35	1.75 ± 0.7	1.71 ± 1.21-1.9	1.74 ± 0.9
	INP	1.56 ± 0.46	1.53 ± 0.5	1.59 ± 0.6	1.75 ± 0.94
eGFR (mL/min/1.73m^2^), median (IQR)	Global	41 (34-50)	35 (28-42)	37 (28-46)	35 (26-44)
	IP	46 (37-57)	32 (27-40)	37 (27-45)	35 (26-44)
	INP	36 (31-43)	38 (31-45)	37 (30-46)	36 (27-45)
ACR (mg/g), median (IQR)	Global	27 (7-179)	36 (7-229)	55 (14-130)	37 (6-335)
	IP	22 (6-171)	34 (8-142)	55 (7-143)	32 (5-520)
	INP	35 (9-189)	61 (5-328)	60 (23-114)	45 (13-238)

ACR, urinary albumin/Creatinine ratio (mg/g); eGFR, estimated glomerular filtration rate by CKD-EPI (mL/min/1.73m2); INP, initial non-progressors; IP, initial progressors; IQR, interquartile range; sCr, serum creatinine (mg/dL); SD, standard deviation.

**Figure 1:**
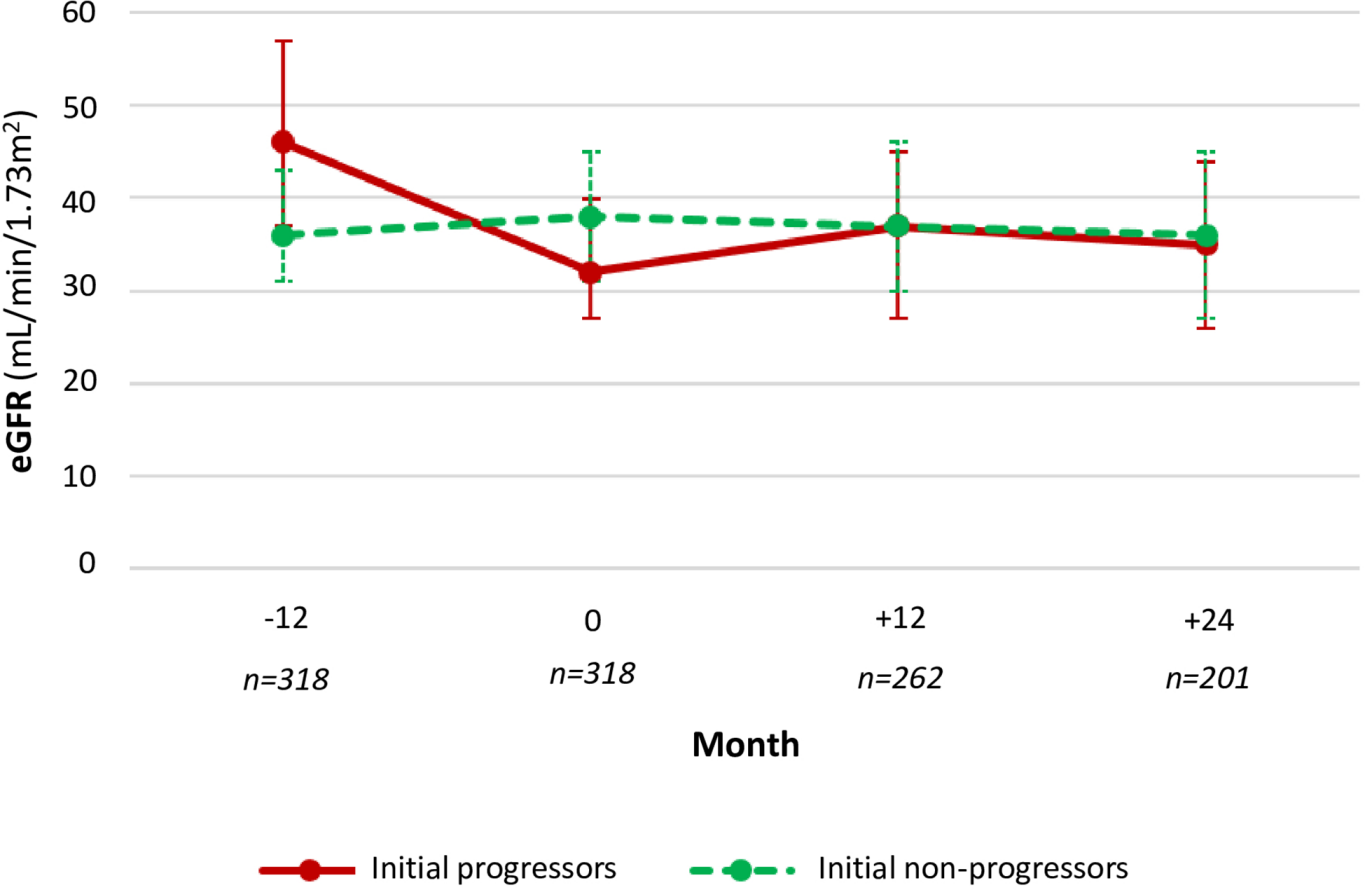
Evolution of the glomerular filtration rate according to initial progression.

Among initial progressors (N=177), eGFR decline before the first consultation was more marked (from 46 [37-57] to 32 [27-40] mL/min/1.73m2). However, eGFR remained stable at 12 and 24-month-follow-up (median 37 [27-45] mL/min/1.73m2 at 12 months and 35 [26-44] mL/min/1.73m2 at 24 months) (**Table 3**). Initial non-progressors (N=141) showed no change in median eGFR between 12 months before (36 [31-43] mL/min/1.73m2) and 24 months after (36 [27-45] mL/min/1.73m2), (**Table 3**; **Figure 1**).

After 12 months of follow-up, 14 patients achieved an eGFR ≤15 mL/min/1.73m2 and 10 patients after 24 months. None required dialysis.

### 3.3 Patient progression

Fifty-five percent (177 of 318 patients) met the definition of initial progressor. 26.3% and 13.4% were progressors at 12 and 24 month-follow up, respectively.

**Figure 2:**
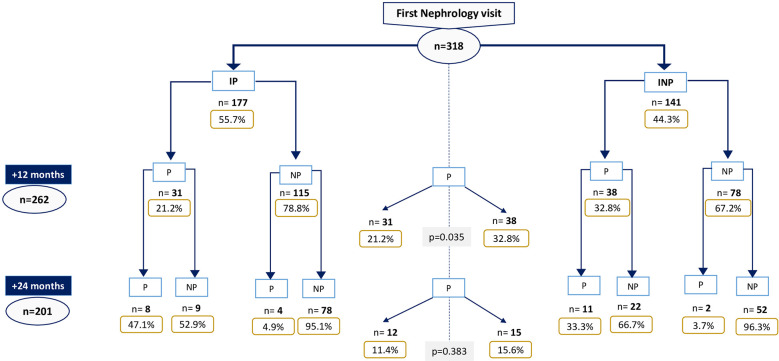
Progressor patients throughout follow-up. IP, Initial-progressors; INP, Initial non-progressors; P, Progressors; NP, Non-progressors.

Within the initial-progressors subgroup, 21.2% (31/146 patients) maintained progression criteria after 12 months, and 11.4% (12/105) after 24 months. Among the initial non-progressors, 32.7% (38/116) became progressors after 12 months and 15.6% (15/96) remained progressors after 24 months (**Figure 2**). After 12 months, initial-progressors showed less progression than initial non-progressors (0.035).

A more significant number of patients received any intervention by the nephrologist in the initial-progressor group (67% of initial-progressors vs. 52,9% of initial non-progressors, p=0.01).

### 3.4 Progression-related factors in all patients

When analyzing factors associated with progression, we found that higher ACR at the time of consultation was related to progression at 12 months (p=0.057). Moreover, patients with higher eGFR and lower sCr progressed more at 12 (p=0.009 and p=0.052, respectively) and 24 months (p=0.0024 and p=0.329, respectively).

In the univariate (UV) and in the MV analysis, the presence of hypertension was associated with progression after 24 months (p=0.009 for UV and p=0.013 for MV) (**Table 4**). Female sex was a protective factor for progression at 24 months (p=0.016). However, it must be taken into account that men presented higher baseline albuminuria (male 89 [19-478] mg/g, female 16 [4-105] mg/g, p<0.001), which seems to act as a confounding factor since after establishing an MV analysis this significance is lost (p=0.108). The rest of comorbidities did not show statistical association.

**Table 4:** Progression related factors after 12 and 24 months in all patients.

n=318	Month +12 (n=262)				Month +24 (n=201)			
Baseline characteristics (first Nephrology consultation)	P (n=69)	NP (n=193)	p (UV)	p (MV)	P (n=27)	NP (n=174)	p (UV)	p (MV)
Female*, n (%)*	33 (47.8)	106 (54.9)	0.311	–	9 (33.3)	101 (58)	0.016	–
Age (years), *mean ± SD*	84.1 ± 3.3	85.1 ± 4.2	0.06	–	84.3 ± 3.4	84.8 ± 4.2	0.564	–
sCr (mg/dL), *mean ± SD*	1.51 ± 0.38	1.69 ± 0.79	0.052	–	1.53 ± 0.4	1.63 ± 0.52	0.329	–
eGFR (mL/min/1,73m^2^), *median (IQR)*	39 (31-45)	35 (28-42)	0.009	–	41 (32-50)	35 (28-42)	0.024	–
ACR (mg/g), *mediana (RIQ)*	65 (14-555)	33 (5-210)	0.057	–	129 (19-791)	34 (5-224)	0.098	–
Charlson index (points), *mean ± SD*	7.3 ± 1.6	7.01 ± 1.9	0.28	–	7.5 ± 1.9	7 ± 1.7	0.182	–
Hypertension, *n (%)*	61 (88.4)	176 (91.2)	0.499	0.262	21 (77.8)	162 (93.1)	0.009	0.013

ACR, urinary albumin/Creatinin ratio (mg/g); eGFR, estimated glomerular filtration rate by CKD-EPI (mL/min/1.73m2); IQR, interquartile range; DM, diabetes mellitus; NP, non- progressor; P, progressor; p (UV), p of univariate analysis; p (MV): p of multivariate analysis; sCr, serum creatinine (mg/dL).

### 3.5 Initial-progressors progression risk factors

Among initial progressors, hypertension was a risk factor for eGFR decrease after 24 months (p=0.016 for UV and p=0.028 for MV). Other factor presenting statistical significance at 12 months in the UV analysis was solitary kidney (p=0.031), although the MV analysis did not confirm this association (**Table 5**). The rest of comorbidities did not show statistical association.

**Table 5:** **Progression related factors after 12 and 24 months in initial progressors**.

n=177	Month +12 (n=146)				Month +24 (n=105)			
Baseline characteristics (first Nephrology consultation)	P (n=31)	NP (n=115)	p (UV)	p (MV)	P (n=12)	NP (n=93)	p (UV)	p (MV)
Female*, n (%)*	13 (41.9)	65 (56.5)	0.148	–	5 (41.7)	52 (55.9)	0.351	–
Age (years), *mean ± SD*	84.2 ± 3.7	85.4 ± 4.6	0.201	–	85.6 ± 3.9	85.1 ± 4.6	0.751	–
sCr (mg/dL), *mean ± SD*	1.68 ± 0.39	1.76 ± 0.78	0.599	–	1.65 ± 0.43	1.72 ± 0.54	0.669	–
eGFR (mL/min/1,73m^2^), *median (IQR)*	35 (25-41)	34 (27-40)	0.839	–	36 (26-43)	33 (27-39)	0.475	–
ACR (mg/g), *mediana (RIQ)*	36 (10-108)	35 (6-180)	0.853	–	31 (13-299)	37 (7-195)	0.841	–
Charlson index (points), *mean ± SD*	7.5 ± 1.8	7 ± 1.9	0.158	–	7.83 ± 2	7 ± 1.9	0.163	–
Hypertension, *n (%)*	26 (83.9)	105 (91.3)	0.226	0.146	9 (75)	88 (94.6)	0.016	0.028
Solitary kidney, *n (%)*	3 (9.7)	2 (1.7)	0.031	0.316	1 (8.3)	0 (0)	0.005	1

ACR, urinary albumin/Creatinin ratio (mg/g); eGFR, estimated glomerular filtration rate by CKD-EPI (mL/min/1.73m2); IQR, interquartile range; NP, non- progressor; P, progressor; p (UV), p of univariate analysis; p (MV), p of multivariate analysis; sCr, serum creatinine (mg/dL).

Among the initia progressors on whom the nephrologist performed some intervention in the first consultation, a lower proportion of progressors was found at 12 months compared to those who did not undergo any intervention (15.8% vs 34.1%, p=0.014).

### 3.6 Initial non-progressors progression risk factors

Patients with better kidney function (higher eGFR and lower Crs) showed more significant progression at 12 (p=0.04 and p=0.023, respectively) and 24 months (p=0.022 and p=0.451, respectively). Increased albuminuria was a risk factor for progression at 12 and 24 months among “initial non-progressors” (p=0.022 and p=0.039, respectively). Female sex was a protective factor for progression at 24 months (p=0.016), although after including this factor with albuminuria in the MV analysis, this significance is lost (p=0.229) (**Table 6**). The rest of comorbidities did not show statistical association.

**Table 6:** Progression related factors after 12 and 24 months in initial non-progressors.

n=141	Month +12 (n=116)				Month +24 (n=96)			
Baseline characteristics (first Nephrology consultation)	P+12 (n=38)	NP+12 (n=78)	p (UV)	p (MV)	P+24 (n=15)	NP+24 (n=81)	p (UV)	p (MV)
Female*, n (%)*	20 (52.6)	41 (52.6)	0.995	-	4 (26.7)	49 (60.5)	0.016	-
Age (years), *mean ± SD*	84 ± 3.1	84.6 ± 3.6	0.39	-	83.3 ± 2.6	84.4 ± 3.5	0.252	-
sCr (mg/dL), *mean ± SD*	1.39 ± 0.32	1.59 ± 0.48	0.023	-	1.44 ± 0.38	1.54 ± 0.49	0.451	-
eGFR (mL/min/1,73m^2^), *median (IQR)*	43 (37-50)	37 (29-44)	0.004	-	48 (39-50)	38 (31-44)	0.022	-
ACR (mg/g), *mediana (RIQ)*	228 (15-1060)	22 (4-220)	0.022	-	271 (53-1036)	25 (4-225)	0.039	-
Charlson index (points), *mean ± SD*	7.1 ± 1.4	7.1 ± 1.7	0.96	-	7.2 ± 1.7	7 ± 1.5	0.628	-

ACR, urinary albumin/Creatinin ratio (mg/g); eGFR, estimated glomerular filtration rate by CKD-EPI (mL/min/1.73m2); IQR, interquartile range; NP, non- progressor; P, progressor; p (UV), p of univariate analysis; p (MV), p of multivariate analysis; sCr, serum creatinine (mg/dL).

No relationship was found between the intervention of the nephrologist and the evolution of renal function among initial non-progressors (31.7% progressors at 12 months among those who underwent intervention vs 32,7% progressors at 12 months among those who did not undergo intervention, p=0.914).

### 3.7 Evolution according to nephrologist intervention

Among initial progressors, nephrologist intervention was associated with a lower proportion of progressors at 12 months (15.8% vs 34.1%, p=0.014). No differences were found depending on whether the decision was to increase or decrease diuretics/other antihypertensives (p=0,933). Among initial non-progressors, this association was not demonstrated.

Median albuminuria did not suffer significant differences according to the intervention of the nephrologist neither in the total nor in the subgroups of initial progressors and initial non-progressors.

### 3.8 Patients with eGFR <30 mL/min/1.73m2

Ninety-eight patients with an eGFR <30 mL/min/1.73m2 were included. Sixty-four (65.3%) were women and with a median age of 84 ± 3.5 years.

The mean sCr was 2.2 ± 0.8 mg/dL, and the median eGFR among these patients was 25 (22-28) mL/min/1.73m2. Median albuminuria was 89 (10-417) mg/g.

#### 3.8.1 Evolution of renal function among patients with eGFR <30 mL/min/1.73m2

eGFR decreased a median of 8 mL/min/1.73m2 until first Nephrology assessment (eGFR 25 [22-28] mL/min/73m2), and it remained stable afterward (24 [18-33] and 25 [18-32] mL/min/1.73m2 at 12 and 24 months, respectively).

Initial progressors (N=66), suffered a more significant initial decrease (from 36 [32-40] to 24 [22-28] mL/min/1.73m2), but the decrease did not progress later on (median eGFR 23 [18 -36] mL/min/1.73m2 after 12 months and 26 [19-36] mL/min/1.73m2 after 24 months). Initial non-progressors (N=32) varied median eGFR by 2 mL/min/1.73m2 between 12 months earlier (28 [24-30] mL/min/1.73m2) and first visit (26 [22-28] mL/min/1.73m2), and maintained similar values throughout the follow-up (after 24 months eGFR 25 [14-31] mL/min/1.73m2).

#### 3.8.2 Progression and risk factors among eGFR <30 mL/min/1.73m2 patients

**Figure 3:**
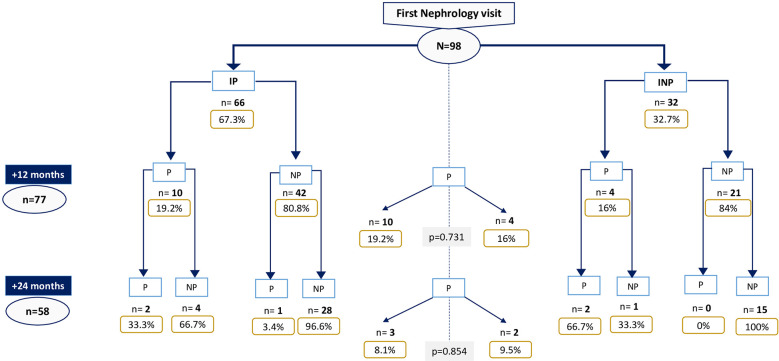
Progressor patients throughout follow-up among those with first Nephrology vist eGFR<30 mL/min/1.73m2. IP, Initial-progressors; INP, Initial non-progressors; P, Progressors; NP, Non-progressors.

Sixty-three percent (66/98) of patients met initial progressor definition. This number fell to 18.2% (14/77) after 12 months and 8.6% (5/58) after 24 months of follow-up (**Figure 3**).

Albuminuria was associated with progression after 12 months (progressors 1075 [394-2117] mg/g vs. non-progressors 75 [9-378] mg/g, p=0.003), and here was a trend towards progression at 24 months, although this did not reach statistical significances, probably due to low statistical power (progressors 872 [264-872] mg/g vs. non-progressors 98 [8-426] mg/g, p=0.059).

There was a benefit of nephrologist intervention among initial progressors with eGFR <30 mL/min/1.73m2 (21.7% progressors at 12 months among those who underwent intervention vs 100% progressors at 12 months among those who did not undergo intervention, p=0.014). No relationship was found between the intervention of the nephrologist and the evolution of renal function among initial non-progressors (11.8% progressors at 12 months among those who underwent intervention vs. 0% progressors at 12 months among those who did not undergo intervention, p=0.569).

## 4 Discussion

According to our data, most of octogenarian patients referred to the Nephrology department will maintain stable renal function after two years of follow-up. Although more than half of our patients (55.7%) met progression criteria when they were evaluated for the first time at Nephrology consultation, this proportion fell to 26% and 13% after 12 and 24 months of follow-up, respectively. When considering patients with ≥4 stage CKD, the results were similar: although the majority (67.3%) were initial progressors, after 24 months of follow-up, this proportion was reduced to 8.6%.

It is crucial to differentiate between the natural progression of renal function in the elderly patient and the actual progression of CKD (3). Recently, a nonlinear GFR decline with aging has been demonstrated with aging, even using cystatin C determinations (15). It must be taken into account that muscle mass decreases at a similar rate to urinary excretion of creatinine, so the mean values of sCr do not vary as markedly as in young people (1, 12). This physiological phenomenon implies that basing the evaluation of renal function according to sCr-depending formulas may not be appropriate for this population (16). For this reason, some studies have decided to measure GFR by other methods in elderly patients, such as iohexol, finding a negative linear association between age and GFR in older patients, even among those without significant comorbidities, the “healthy elderly” (17). In addition, renal mass suffers a decrease of 10% per year in people over 40 years old, and this process takes place at the cost of cortical thinning and loss of nephrons (3), which would support a non-pathological origin of the decrease in eGFR in the elderly.

Proteinuria is a known CKD and cardiovascular mortality-related factor (1, 7, 9, 18). In the subgroup analysis, we found that among initial non-progressors, there was a significant relationship between higher ACR and transformation to progressor at 12 and 24 months. This factor was not confirmed in the initial progressors, possibly due to more controlled albuminuria from the beginning (more incisive GP).

Our results suggest that optimizing proteinuria is essential from an early stage, even when there is no marked eGFR decline. It must be noted that only 18.9% of the patients had an ACR determination the year prior to the Nephrology referral; therefore, it is necessary to insist on the importance of this value among GP.

The association between higher baseline eGFR and risk of progression is partially explained by the correlation curve between sCr and GFR. The lower the GFR gets, the less sCr variations affect it (19). However, that does not explain the trend towards a lower risk of progression among those with a higher sCr that has been observed too (although with less robustness), which could be attributed to a closer follow-up of these patients or regression to the mean.

Gender is another factor studied as a predictor for progression, for which there are conflicting conclusions (4, 13, 20). In our study, male sex represented a risk factor for progression at 24 months. However, men had greater baseline albuminuria, acting as a confounding factor since this association disappeared in the MV analysis.

Different studies establish a relationship between kidney disease and comorbidities such as hypertension, DM, or HF (4, 5, 7, 9). In the present study, it was challenging to find a solid correlation between the different comorbidities studied and risk of progression. However, hypertension emerged as a possible risk factor at two years, as it has already been established in other studies focused on the elderly population (21).

While CKD’s prevalence diagnosis increases, the proportion of elderly patients who develop terminal CKD is lower than in younger population groups (1, 5, 6). Moreover, many elderly patients do not die from terminal renal insufficiency but mainly from cardiovascular pathologies (11). Authors such as Locatelli F et al. and Mora-Gutiérrez JM et al., emphasize that the central axis of the management of the elderly patient must be the control of the cardiovascular risk factors more than the strict control of the eGFR (5, 6).

In our cohort, the nephrologist intervention proved to be a protective factor for progression among initial progressors, so special attention should be paid to these patients since their early referral seems to slow their impairment (in fact after 12 months, initial-progressors showed less progression than initial non-progressors). No differences were found depending on whether the decision was to increase or decrease diuretics/other antihypertensives, an individualized approach should be applied to each patient. It is important to highlight that initial non-progressors did not benefited from neprhological intervention, which implies that GP could probably follow those with an annual eGFR decline ≤5 mL/min/1.73m2.

There are telematic tools that may be useful for better communication between GP and specialists, and which can be applicable to this group of patients who do not clearly benefit from nephrologist evaluation. In our hospital e-consultations have been incorporated in the last years as part of our clinical practice. This consists of an interactive web dialogue through which GP can raise their doubts about patients who are or are not followed previously in our department, avoiding unnecessary referrals and providing confidence to the GP in the management of kidney diseases.

Our study presents the limitations resulting from the partial retrospective collection of data. In addition, this is a single-center study with a limited number of patients; thus, conclusions may not apply to all population groups. The strengths of this real life clinical study lie in the homogeneity of the population studied and the existence of statistically significant results, consistent with other studies, which can serve as a tool in the daily clinical practice of Primary Care consultations. Applying stricter criteria for referral to Nephrology in this group of octogenarian patients could entail fewer unnecessary consultations, with essential repercussions on social, economic, and health aspects.

## 5 Conclusions

In our study, octogenarian patients who have stable renal function (eGFR decline ≤5 mL/min/1.73m2) and no proteinuria kept stable renal function after 24 months of follow-up and did no benefit from a nephrologist’s assessment. We suggest that these patients should be followed by their GP with optimization of functional factors and proteinuria control and only be referred when the authentic progression of renal failure is confirmed (rather than considering an isolated value). Proteinuria is a main risk factor, so it is essential to generalize the use of ACR determination in Primary Care.

## Data availability statement

The raw data supporting the conclusions of this article will be made available by the authors, without undue reservation.

## Ethics statement

The studies involving human participants were reviewed and approved by i+12 Hospital 12 de Octubre. Written informed consent for participation was not required for this study in accordance with the national legislation and the institutional requirements.

## Author contributions

EM, AF and FV designed the study. AF, FV, TC, JSe, FG, EH, EG and PA collected data. AF and FV analyzed and interpreted the data. AF and EM wrote the manuscript. IZ, JP, JSa, RB, MR and LC revised the manuscript. All authors contributed to the article and approved the submitted version.
